# Structure–Function Analysis in Macular Drusen With Mesopic and Scotopic Microperimetry

**DOI:** 10.1167/tvst.9.13.43

**Published:** 2020-12-28

**Authors:** Giovanni Montesano, Giovanni Ometto, Bethany E. Higgins, Costanza Iester, Konstantinos Balaskas, Adnan Tufail, Usha Chakravarthy, Ruth E. Hogg, David P. Crabb

**Affiliations:** 1 City, University of London—Optometry and Visual Sciences, London, UK; 2 NIHR Biomedical Research Centre, Moorfields Eye Hospital NHS Foundation Trust and UCL Institute of Ophthalmology, London, UK; 3 Centre for Public Health, Queen's University Belfast, Belfast, Northern Ireland

**Keywords:** drusen, structure–function, microperimetry, optical coherence tomography, age-related macular degeneration

## Abstract

**Purpose:**

To investigate the structure–function relationship in eyes with drusen with mesopic and scotopic microperimetry.

**Methods:**

We analyzed structural and functional data from 43 eyes with drusen. Functional data were acquired with mesopic and scotopic two-color (red and cyan) microperimetry. Normative values were calculated using data from 56 healthy eyes. Structural measurements were green autofluorescence and dense macular optical coherence tomography scans. The latter were used to calculate the retinal pigment epithelium elevation (RPE-E) and the photoreceptor reflectivity ratio (PRR). The pointwise structure–function relationship was measured with linear mixed models having the log-transformed structural parameters as predictors and the sensitivity loss (SL, deviation from normal) as the response variable.

**Results:**

In the univariable analysis, the structural predictors were all significantly correlated (*P* < 0.05) with the SL in the mesopic and scotopic tests. In a multivariable model, mesopic microperimetry yielded the best structure–function relationship. All predictors were significant (*P* < 0.05), but the predictive power was weak (best *R*^2^ = 0.09). The relationship was improved when analyzing locations with abnormal RPE-E (best *R*^2^ = 0.18).

**Conclusions:**

Mesopic microperimetry shows better structure–function relationship compared to scotopic microperimetry; the relationship is weak, likely due to the early functional damage and the small number of tested locations affected by drusen. The relationship is stronger when locations with drusen are isolated for the mesopic and scotopic cyan test.

**Translational Relevance:**

These results could be useful to devise integrated structure–function methods to detect disease progression in intermediate age-related macular degeneration.

## Introduction

Macular damage and its progression can be assessed with functional and structural tests in patients with intermediate age-related macular degeneration (iAMD). Two commonly used technologies for structural evaluation are spectral domain optical coherence tomography (SD-OCT) and fundus autofluorescence (FAF).^1^ Compared to color fundus photography, they provide data that are more easily quantified, such as thickness and reflectivity of different retinal layers with SD-OCT and fluorescent properties of lipofuscin with FAF.^1^ In iAMD, SD-OCT has been employed to identify location, characteristics, and size of macular drusen, a predominant structural feature in these patients,^2^^,^^3^ and both OCT and FAF have been used to aid detection of progression to geographic atrophy (GA).^4^

Structural changes define the disease, but visual function is more important to the patient. Moreover, functional testing is also of paramount importance in detecting the progression of damage.^5^^–^^8^ Several functional tests are available, including best-corrected visual acuity (BCVA), dark adaptometry, reading speed, and microperimetry.^7^^,^^9^^–^^11^ The latter is a perimetric test that employs continuous infrared fundus imaging to track and compensate for eye movements during the test.^12^^–^^14^ Originally introduced to allow perimetry in patients with advanced macular damage and poor fixation, it is presently a cornerstone in characterization of functional loss in AMD due to its better correlation with anatomic features compared to traditional perimetry and global functional metrics such as BCVA.^13^^–^^16^ Moreover, microperimetry is an approved functional outcome for clinical trials by the US Food and Drug Administration.^17^ Microperimetry has the potential to highlight differences in function in iAMD when performed in mesopic and scotopic conditions,^15^^,^^18^^–^^20^ since the visual performance of these patients is affected by lighting conditions.^21^^–^^23^

There exists interest in developing outcome measures with sufficient precision to assess change in iAMD for clinical trials, as interventions are more likely to be effective if given early in the evolution of AMD before the onset of GA, which represents an advanced stage of atrophy and permanent structural damage. Moreover, clinical trials with efficient end points are a critical step for speeding up the process of proving effective treatments for patient benefit.^24^ It is not sufficient to test structural and functional endpoint categories in isolation. Detecting progression might be limited by the imprecision in both the functional and structural measurements. Therefore, a natural step forward is to combine these two different sources of information. The starting point of this work is to examine the level of association between structural and functional measures in iAMD. Since microperimetry provides accurate anatomic landmarks, a more precise mapping of perimetric sensitivities onto the structural maps is possible.

The localized (pointwise) relationship between structure and function in iAMD has been studied before, in both mesopic^16^^,^^20^ and scotopic^20^ conditions. However, the only direct comparison of the structure–function relationship between mesopic and scotopic microperimetry has been performed with the Nidek MP-1S microperimeter (Nidek Technologies, Padua, Italy), which uses achromatic stimuli for both the mesopic and scotopic test.^20^ In contrast, a recent version of the MAIA microperimeter (CenterVue, Padua, Italy), the scotopic MAIA (S-MAIA), is able to stimulate the retina with two different wavelengths, potentially targeting different pathways in the dark adapted retina.^15^^,^^25^ Comparing the structure–function relationship in these different testing conditions could identify candidate paradigms to characterize progression in iAMD, and this is the main idea of our work.

We aim to test the strength of the relationship between structural features of drusen with microperimetric sensitivities at individual test locations acquired with achromatic stimuli in mesopic conditions and with two wavelength stimuli in scotopic conditions.

## Methods

### Data Set

We used data prospectively collected in the Northern Ireland Sensory Aging 2 (NISA-2) study (https://clinicaltrials.gov/ct2/show/NCT02788695), consisting of a subset of participants in the Northern Ireland Cohort for the Longitudinal Study of Ageing (NICOLA) asdas study (https://www.qub.ac.uk/sites/NICOLA/), which is an ongoing population-based study conducted at Queen's University, Belfast. All data were collected after acquiring written informed consent from the participants, and the study adhered to the tenets of the Declaration of Helsinki. The objective of NISA-2 was to obtain imaging and functional data from an aging population (*N* = 402). For the current study, we extracted the data pertaining to a subcohort of participants without diabetes mellitus who had SD-OCT macular scans, dual-wavelength FAF images, and scotopic and mesopic microperimetry. Scotopic perimetry was only available during the latter part of data collection, and the earliest versions of the commercial device were used. Only one eye per patient was tested for microperimetry in this study. Prior to imaging and microperimetry, all patients were pharmacologically dilated with tropicamide 1%. The study eye was chosen as the one with worse BCVA. If both eyes were eligible, the right eye was chosen. History of amblyopia precluded the selection of the amblyopic eye. Two independent graders first evaluated all eyes using color fundus pictures. These were classified into different AMD groups according to the Beckman classification,^3^ reported in the Appendix. Only people with advanced AMD (group 4 in our classification) were excluded based on this classification. The same graders then evaluated the SD-OCT images to identify morphologic abnormalities of the outer retina. We used this classification to detect eyes with any macular drusen and healthy controls. Of course, drusen do not necessarily identify people with iAMD. However, 33 of 43 (77%) people with drusen identified on OCT scans were classified as having iAMD with the Beckman classification. Eyes with reticular pseudodrusen were excluded. Eyes with pseudodrusen were identified using color fundus pictures, infrared scanning laser ophthalmoscopy (SLO), or FAF, regardless of the SD-OCT appearance. All images were further screened by an ophthalmologist (GM) to identify and exclude eyes with diseases of the inner retina, looking for localized or diffused thinning of the retinal nerve fiber layer or the ganglion cell layer. Eyes with poor-quality OCT scans or for which FAF and both mesopic and scotopic tests were not available were also excluded. Poor-quality scans were those in which the retinal thickness was not fully contained within the image or graders were not able to correctly identify all retinal layers. This led to the final selection of 56 visually healthy controls and 43 people with drusen. Demographic characteristics of the sample are reported in Table 1. Four eyes with drusen had an intraocular lens implant. Lens opacity in the remaining eyes was graded with a Pentacam Scheimpflug System (Oculus, Wetzlar, Germany) using the Pentacam Nucleus Staging (PNS) classification.^26^ Twenty-three eyes with drusen and 38 healthy eyes had a PNS = 0. The remaining eyes had a PNS = 1, except for one eye with drusen with a PNS = 2. No posterior capsular opacity was reported.

**Table 1.** Demographics of the Analyzed Sample

		Median [Interquartile Range]	
Characteristic		Healthy (*n* = 56)	Drusen (*n* = 43)
Age (y)		62 [58, 67]	72 [64, 78]
Male/female, No.		26/30	22/21
BCVA (letters)		88 [84, 90]	84 [79, 87]
Mean sensitivity (dB)	Mesopic	25.48 [24.58, 26.20]	24.32 [22.77, 25.25]
	Scotopic, red	13.23 [12.24, 14.03]	12.53 [10.40 14.07]
	Scotopic, cyan	11.74 [10.50, 12.42]	10.43 [9.12 12.10]
Spherical equivalent (diopters)		0.5 [−1.12, +1.50]	0.5 [0, +1.62]

### SD-OCT Imaging

The SD-OCT data used in this analysis were composed of macular scans covering the central 30 × 30 degrees with 61 B-scans (Automatic Real-time Tracking (ART) set to nine-scan average). All data were collected with the Spectralis HRA–SD-OCT device (Heidelberg Engineering, Heidelberg, Germany). The device uses infrared SLO to track eye movements during the acquisition of the image. As a result, all OCT maps can be overlaid with the infrared fundus picture. The image acquisition was performed with a central fixation target and focusing on the retina. The average quality index of the OCT scans was 32.01 ± 2.82 dB (mean ± SD) for patients with drusen and 32.68 ± 2.28 dB for the healthy cohort.

### FAF Imaging

The Spectralis HRA–SD-OCT equipped with a macular pigment optical density (MPOD) module is able to acquire dual-wavelength autofluorescence (AF) data. For the acquisition, fluorescence is generated by exciting the retina with two different wavelengths, blue autofluorescence (488 nm) and green autofluorescence (GAF, 518 nm). They both excite lipofuscin, the prominent naturally occurring fluorophore in the retina, mainly localized at the level of the retinal pigment epithelium (RPE). However, the blue light is absorbed much more than the green light by the macular pigment (MP), concentrated at the fovea in the inner layers. The fluorescent signal was recorded at a wavelength above 530 nm, filtering out the excitation wavelengths, after bleaching the retina for 30 seconds to minimize rhodopsin absorption. The combination of the signal from the two wavelength provides an optical quantification of the macular pigment density (see later). Also, the image generated with 518-nm excitation provides images that are minimally affected by MP masking and are used for our structure–function analysis (see later).

### Microperimetry

For both the mesopic and the scotopic test, a Goldmann III size (0.43 degrees diameter) was used. A baseline infrared image was acquired and used as a reference for tracking during the whole test. The testing grid was centered on the preferred retinal locus (PRL) of fixation, determined at the beginning of the test with a short fixation task (10 seconds) using a fixation target of 1 degree in diameter. This meant that the center of the grid might not coincide with the anatomic fovea. The grid was composed of 44 testing locations along fixed meridians (see  later) plus a central testing location and one on the optic nerve head; these two locations were excluded from the analysis (the central location is tested as a separate foveal location and not tracked). The tests were preceded by a practice run with the “Fast” protocol to minimize learning effect.

Mesopic microperimetry was performed using a 4-2 staircase strategy in a dark room (ambient light 0.01 lux). No adaptation was performed prior to this test. The mesopic test has a dynamic range of 36 dB. The scotopic testing used the same grid and PRL assessment procedure but used a 2-1 staircase procedure and two wavelengths for the stimulus, cyan (505 nm) and red (627 nm). Prior to scotopic testing, all patients were dark adapted for 30 minutes. The patient was sat comfortably in the dark room, and all possible light sources were covered. A red filter was used for the S-MAIA screen to minimize stray light. The version of the S-MAIA used in this study had a dynamic range of 20 dB. We did not apply any performance-based exclusion criteria for the microperimetric tests, requiring only that the test was brought to completion.

### Structure–Function Analysis

The comparison of the structure–function relationship with different microperimetric tests was performed only on eyes with macular drusen. The data from healthy controls were used only to build a normative reference for perimetric data. The steps for the analysis are reported below. Both structural and functional data were acquired on the same date. The mesopic test was always performed before the scotopic tests (minimum interval 40 minutes). Imaging was performed before the microperimetric tests in 18 of 56 healthy individuals and 35 of 43 people with drusen, with a minimum interval of 20 minutes between imaging and the first microperimetric test (mesopic).

#### SD-OCT Image Analysis

**Figure 1.**
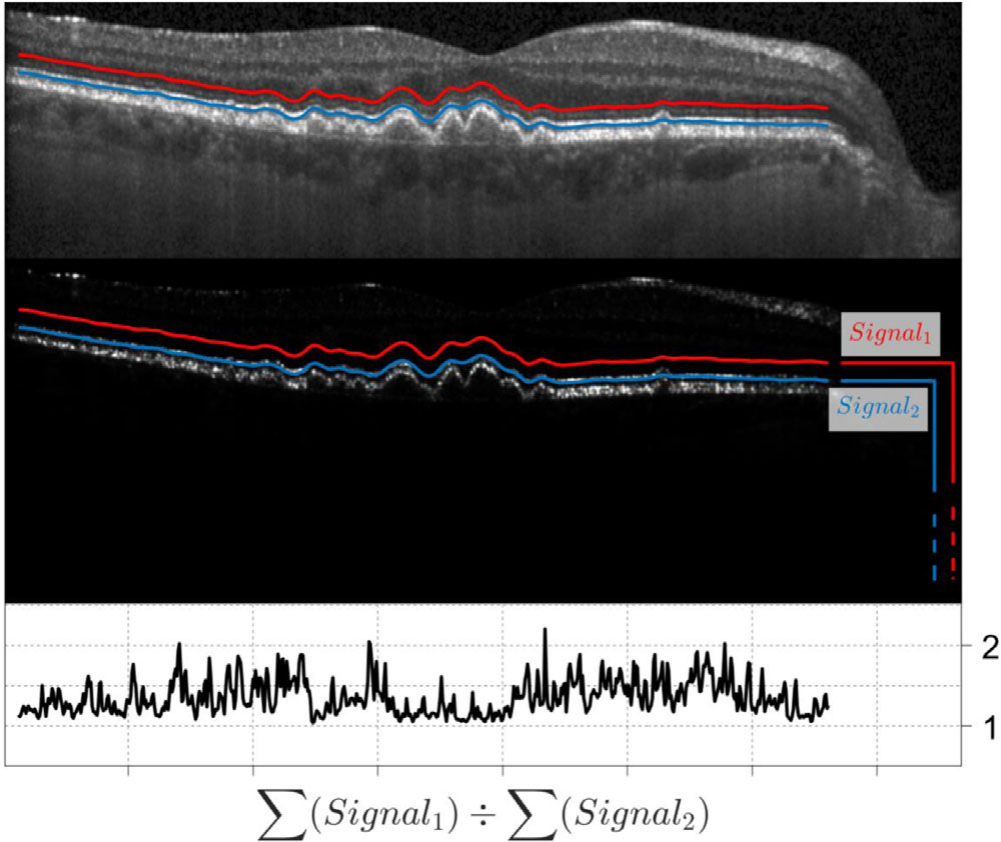
Example of the calculation of the photoreceptor reflectivity ratio (PRR) for a single B-scan. In short, *Signal_1_* and *Signal_2_* are calculated for each A-scan as the linear sum of all the values from the raw OCT image (*middle panel*) below the corresponding profile (*red* for *Signal_1_*, *blue* for *Signal_2_*). The ratio of the two profiles is then computed to obtain the PRR (*bottom panel*). Notice how the PRR value decreases when the reflectivity of the photoreceptor layer is reduced. The two profiles are reported on the scaled image (*top panel*) for reference.

**Figure 2.**
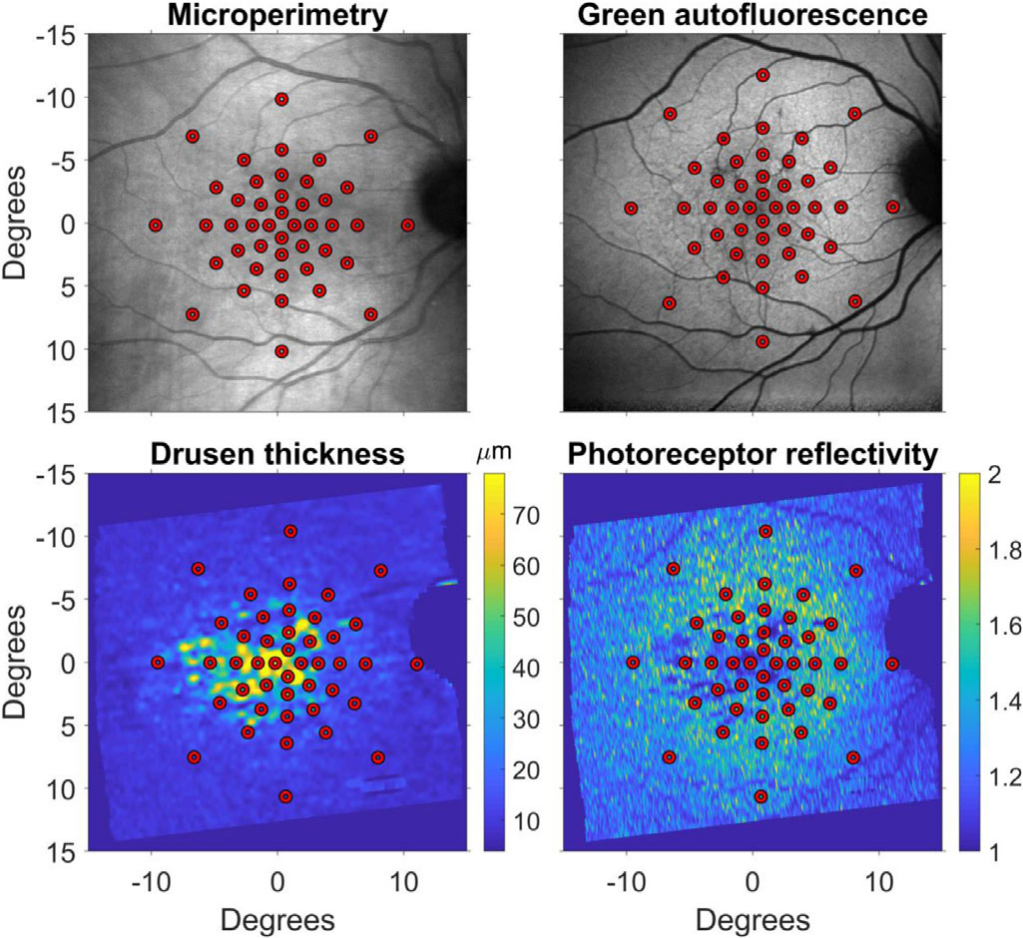
Example of how the tested locations were reported from the fundus image used to track the eye during microperimetry (*top-left*) onto each structural map used for this analysis. Notice how the pattern of photoreceptor reflectivity is similar to that of drusen thickness but does not completely overlap.

All scans were inspected using the Heidelberg Eye Explorer software. The segmentations for the inner limiting membrane and Bruch's membrane (BM) were manually corrected where necessary. The latter was particularly relevant for eyes with drusen, in which the segmentation for the BM was often incorrect and some degree of manual correction was needed in all volumes. The segmentation for the RPE in healthy controls was generally accurate, and minor errors were manually corrected (in 10/56 volumes). In eyes with drusen, an additional step was necessary to accurately segment the RPE, whose elevation was used as a surrogate of drusen thickness. This additional processing was performed in MATLAB (MathWorks, Natick, MA, USA). For all participants, we exported the images from the Spectralis in RAW format (.vol). These files contain segmentation data and the untransformed (linear) OCT scans in single precision. For each A-scan, the intensity profile above the manually segmented BM was normalized by the maximum value in the profile. Then, the first normalized peak above a threshold of 0.4 closest to the BM was used as an initial guess for the location of the RPE. The final segmentation was produced by a customized version of a previously published algorithm,^27^ starting from the initial guess. The segmentation results were visually inspected and corrected where necessary. Some degree on manual refinement was performed in at least one section in all volumes to obtain the best result. This segmentation was used to derive two structural maps (Fig. 2). The first was the RPE elevation (RPE-E), obtained by simple difference between the RPE and BM segmentation. The second was a photoreceptor reflectivity ratio (PRR) map. This was meant to quantify the reflectivity of the photoreceptor outer segments. For this calculation, we assumed the amount of light from the OCT beam reaching a certain depth in the image could be calculated by a linear sum of all the values in each A-scan from that specific depth to the bottom of the image (Fig. 1^2^). A map of this value was calculated twice starting at 6 pixels and 20 pixels above the RPE segmentation for all the B-scans. The ratio of the two maps provided the fraction of light back-reflected in the path across the photoreceptor outer layers. Therefore, a dimmer photoreceptor outer layer would make this ratio close to 1. This calculation was performed on the untransformed images in single precision (Fig. 1).

#### Alignment of Fundus Images

Fundus images from the MAIA were aligned with the infrared SLO fundus images from the Spectralis. The alignment needed to be performed once for the mesopic test and once for the scotopic test, for a total of 198 image pairs. An affine or projective transformation was used. The alignment was performed by a custom software written in MATLAB. All alignments were visually inspected. All failed alignments were identified and a new attempt was made with manually placed landmarks and a projective transformation. A satisfactory alignment (as evaluated by a grader, GM) could be obtained in all the images, but manual landmarks were necessary in 58 of 86 pairs for eyes with drusen and 67 of 112 in the healthy cohort . This alignment was used to report the tested locations onto the structural maps (OCT or FAF) and obtain colocalized measurements within the tested areas, one for each location. FAF images were aligned with the corresponding infrared SLO images from the Spectralis, so that the same spatial transformation for MAIA data could be used for both FAF and infrared SLO images. For this second step, the automated alignment was more often successful, requiring manual intervention in 19 of 99 pairs. The values were calculated as averages within the tested areas. The GAF map was used for these calculations to avoid the effect of MP absorption.

#### Compensation for Macular Pigment Absorption

Sensitivity measured with the cyan stimulus showed a marked decrease toward the foveal center, even in healthy patients. Since the wavelength used for this stimulus is thought to mainly target rods,^25^ this behavior is consistent with the decreasing rod density toward the fovea.^28^ However, the same wavelength is also partially absorbed by the MP, reducing the measured sensitivity. Since MP density can vary across different individuals and is known to be reduced people with AMD,^29^ we developed a method to correct the sensitivities for MP absorption. We calculated a map of the MPOD as the ratio between the blue and the green FAF signal, the latter being minimally absorbed by the MP.^30^^,^^31^ The MP absorption at 505 nm (the wavelength of the cyan stimulus) is approximately 66% of that at 488 nm (interpolated from the values provided by Snodderly et al.).^32^ Therefore, the final compensation for the sensitivity (in logarithmic scale) is

$$\begin{array}{c} C_{dB}=10\;*\;log_{10}\left(0.66\;*\left(MPOD-1\right)+1\;\right), \end{array}$$

where *C_dB_* is the correction in dB that needs to be added to the measured sensitivity. This formula assumes that the macular pigment absorption of the GAF signal is negligible. The result is 0 when the MPOD ratio is equal to 1 (no absorption). This calculation conveniently produces a map whose average value can be calculated for each individual tested location, as above. All negative average values (MPOD ratio <1) were set to zero.

#### Normative Reference for Microperimetry

**Figure 3.**
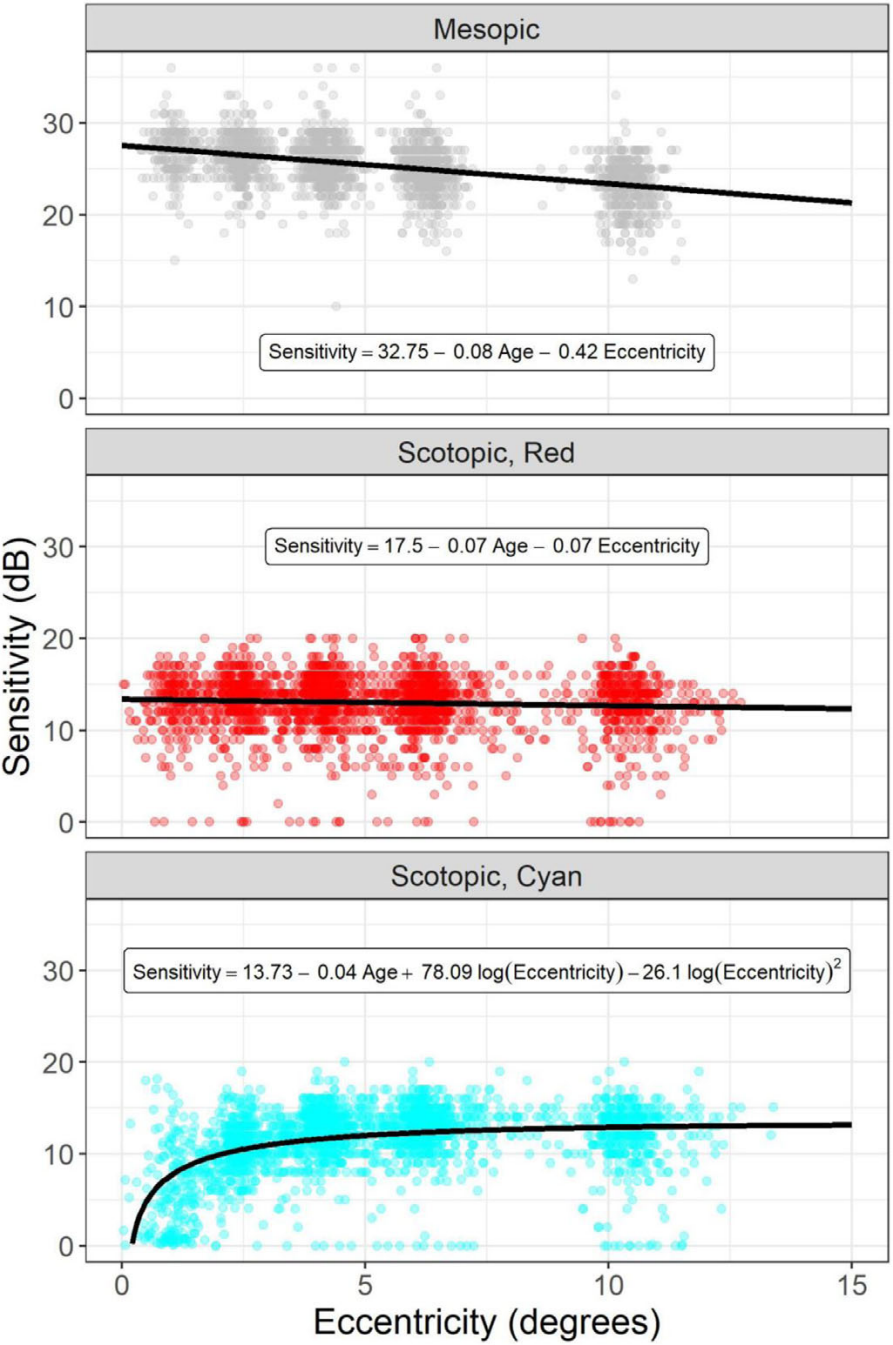
Normative values for the three tests. The *black lines* show the fit of the normative equation (equations reported as text in each graph). In the equations, eccentricity is in degrees and age is in years. The sensitivities for the *cyan* stimulus are compensated for macular pigment absorption. Each point represents a single tested location.

When evaluated in normal controls, age and eccentricity had very different impact on the sensitivity measured with the mesopic and the two scotopic tests. Therefore, to make tests comparable, we built three normative linear models using the data from the healthy controls. In these models, the effect of age was significant (*P* < 0.05) for the mesopic and scotopic red test but not for the scotopic cyan test (*P* = 0.35). The effect of eccentricity could be modeled with a linear relationship for the mesopic and scotopic red test. Its effect was much smaller for the scotopic red test than for the mesopic test (Fig. 3). Consistent with the known change in rod density, the scotopic cyan test showed a marked decrease in sensitivity toward the fovea,^28^ which was retained after correction for MP absorption. Therefore, the change for this test with eccentricity required a more complex adjustment. A good fit was provided by log-transforming the eccentricity and fitting a quadratic polynomial. The normative equations for the three tests for completeness are reported in Figure 3. These models used a random effect on the intercept for the eye. However, we used only the prediction from the fixed effects as a normative reference, which is relevant to the next step for the analysis.

#### Statistical Analysis and Structure–Function Modeling

For our structure–function modeling, we used the sensitivity loss, calculated as a difference between the observed sensitivity and the expected values from the normative models. This allowed us to use the exact same structure–function modeling for all three tests without further correction for eccentricity and age. This was especially useful to isolate the effect of structural parameters.

The structural measurements were not corrected for ocular magnification. This was a deliberate choice, since all our calculations were performed in visual degrees. Ocular magnification due to axial length only affects the apparent lateral size of objects on the retina but has no effect on axial measurements,^33^ such as thickness. For example, the G-III stimulus used for the microperimetric test would have the same angular size regardless of axial length. For our structural analysis, the area covered by the stimulus was then calculated based on the known angular size of the SLO image from the Spectralis (30 × 30 degrees, 768 × 768 pixels) and the known diameter of the stimulus (0.43 degrees).

We used mixed-effect models with a random effect on the intercept to account for correlations among sensitivities measured on the same eye. The three structural predictors (RPE-E, PRR, and GAF) were log_10_-transformed prior to analysis to match the scale of the sensitivity loss. We separately tested the effect of each parameter with univariable models and their combined effect with a multivariable model with full interaction terms. The *R*^2^ values were calculated using the squared residuals from the fixed-effect predictions divided by the variance of the sensitivity values. All calculations were performed in R (R Foundation for Statistical Computing, Vienna, Austria).

## Results

### Univariable Analysis

*R* ^2^ values and *P* values for the univariable analysis are reported in Table 1. Both OCT predictors were significantly related to sensitivity in the mesopic and scotopic tests. The relationship was negative for RPE-E and positive for PRR. GAF was also significantly positively correlated with sensitivity in all tests (*P* < 0.001). Despite very significant *P* values, the correlation was generally weak. The PRR was the strongest predictor for all tests. Overall, the mesopic test showed the highest *R*^2^ values, with the exception of the PRR for the cyan test, which was the highest (Table 1). Individual equations are reported in the Appendix.

GAF was positively but very weakly correlated with PRR (*R*^2^ = 0.003, *P* < 0.001) and negatively correlated with RPE-E, with a stronger relationship (*R*^2^ = 0.02, *P* < 0.001). RPE-E and PRR were also significantly but weakly correlated (*R*^2^ = 0.010, *P* = 0.015).

### Multivariable Analysis

**Figure 4.**
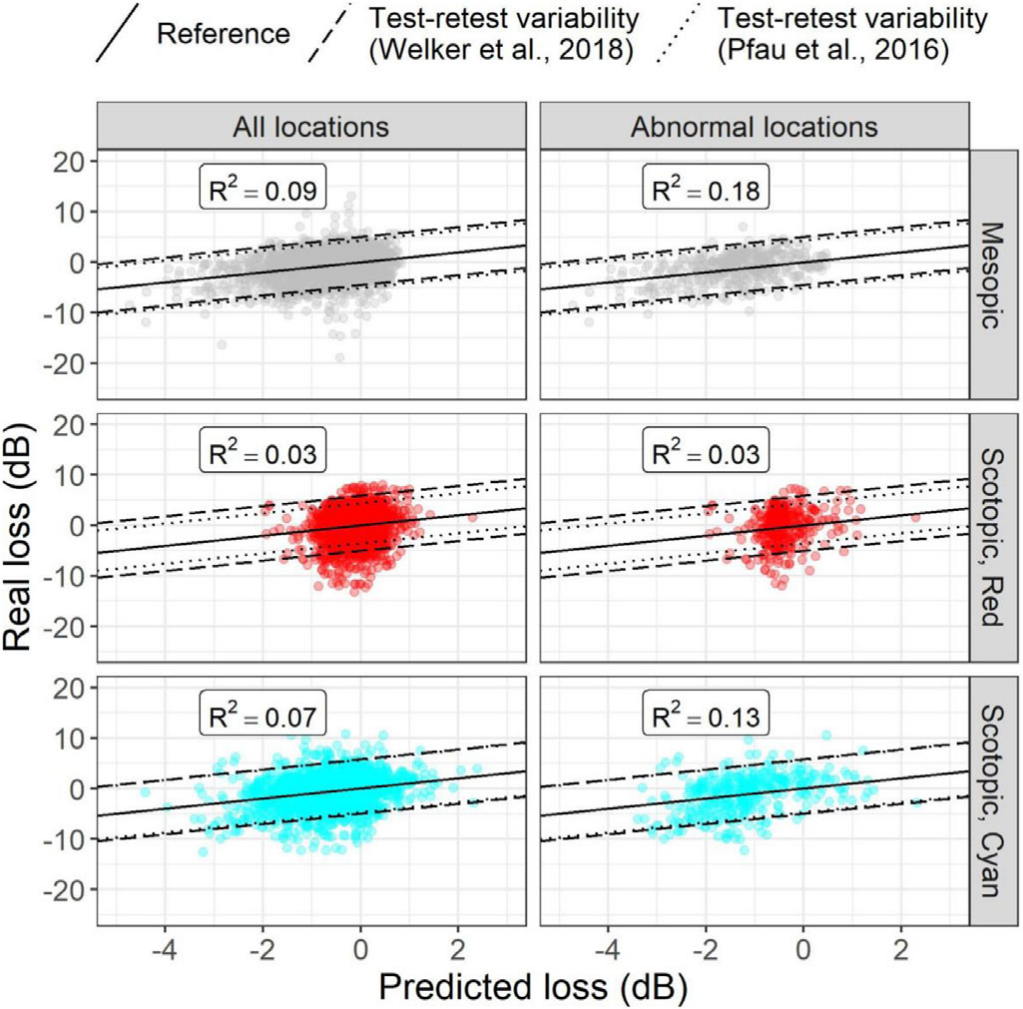
Predictions from the multivariate models. The *R*^2^ for the fixed-effect prediction is reported for each prediction. Abnormal locations are defined as having a drusen thickness >95th percentile of the values from the healthy cohort. Welker et al.^15^ reported data from patients with iAMD but used a device with an extended dynamic range. Pfau et al.^10^ used the same device as in this work but only tested healthy individuals.

The final structure–function model used all structural predictors, including their interactions. The relationship was generally weak (Fig. 4). As for the univariable analysis, the strongest relationship was obtained with the mesopic test. The scotopic red test showed the weakest structure–function relationship. A detailed report of the coefficient values and relative *P* values of the models can be found in the Appendix. When compared to a reduced model that excluded each of the structural predictors and their interactions, all structural parameters significantly improved the fit for the mesopic and scotopic tests (likelihood ratio test, *P* < 0.001, for all parameters except RPE-E for the red scotopic test, for which *P* = 0.021). Of notice, most of the interactions were not significant in the model for the mesopic test (see Appendix). A stepwise model reduction procedure using the Akaike information criterion^34^ yielded a much simpler model, which contained all structural predictors and only the two-way interaction between RPE-E and PRR. However, the three-way interaction was significant for the scotopic tests. Therefore, the full model was retained in all cases for fairness of comparison.

Since most of the tested locations were not affected by drusen, we repeated the analysis only including the locations with abnormal RPE-E values, defined as being larger than the 95th percentile of RPE-E measured in healthy controls. The structure–function relationship was improved for all tests, with an average 2.1-fold increase for the mesopic test, a 1.1-fold increase for the scotopic red test, and a 1.8-fold increase for the scotopic cyan test. However, the relationship remained extremely weak for the scotopic red test. The plots in Figure 4 report predictions from the models along with the 95% test–retest limits of agreement reported by Welker et al.^15^ and Pfau et al.^10^

## Discussion

Our overarching goal was to identify candidate measures that could be integrated to improve the detection of localized progression for both individual patients and clinical trials. We evaluated different structural and functional parameters to detect changes to explore their relationship in people with macular drusen, the prominent structural feature in these patients. We compared the strength of the structure–function relationship between microperimetric tests, mesopic and scotopic, and OCT and FAF measurements. In contrast to previous reports,^20^ we used a two-wavelength scotopic test, which has the potential to target different pathways in the retina.^15^^,^^25^^,^^35^ Moreover, we concentrated our analysis on the predictive ability of the structural parameters. This is essential to assess their usefulness for detection of disease progression.

The strongest relationship in the multivariate analysis between morphologic markers and retinal sensitivity was seen with the mesopic test. The scotopic tests appeared to be limited by a much smaller dynamic range. This can be clearly seen in Figure 3, where the sensitivity values are much lower in both scotopic tests, even in healthy individuals. One explanation for such a difference is the use of a S-MAIA device with a 20-dB testing range. This was the same range as in Saßmannshausen et al.^20^ and Pfau et al.^10^ A more recent version of the device extended the testing range to 36 dB,^15^^,^^35^ and this will likely improve the structure–function relationship. However, we did not find, even in healthy individuals, a clear saturation (ceiling effect) of the measurements, with only a few locations hitting the 20-dB limits. Instead, most of the values were below 15 dB, making a floor effect a more important factor in our results. In this context, it was notable that sensitivity values in healthy individuals and patients with drusen observed in our study were lower than those reported by Welker et al.,^15^ who used a S-MAIA device with a 36-dB dynamic range. However, the sensitivities cannot be directly compared between the two devices, since the stimulus intensities corresponding to the 0- to 20-dB range in our S-MAIA map to 10 to 30 dB in the new device. This also explains why a floor effect was evident even in healthy eyes. Similar results were reported by Pfau et al.,^10^ who assessed test–retest variability in healthy eyes using a S-MAIA with our same dynamic range.

Among the tests we used in determining structure–function relationships, we found that the scotopic red test performed poorly, with a small prediction range. The scotopic cyan test performed better and just marginally worse than the mesopic test. However, the normative profile for the latter required more complicated modeling, due to the peculiar dropoff in sensitivity toward the fovea and its specific wavelength also required an adjustment for MP absorption. This is important, since MP concentration can vary across individuals and is reduced in patients with AMD,^29^ creating differences in the intensity of light that reaches the retina. For our analysis, we derived our correction from an independent optical analysis of blue light absorption, which might not be available in all clinical contexts. The strongest relationship was seen with the mesopic test. This was likely due in part to its extended dynamic range toward lower sensitivities. A secondary analysis on locations with abnormal (thicker) RPE-E values revealed that most of the locations likely affected by drusen were correctly predicted to have lower sensitivities (Fig. 4). These are the locations in which progression is more likely to be observed in a longitudinal follow-up, making this result particularly valuable, and this is noteworthy. This was also the case for the scotopic cyan test but not for the scotopic red test. In general, however, the predictive power of the structure–function models was small. This was unsurprising, since the sensitivity loss in patients with drusen (not necessarily iAMD) is expected to be small^15^^,^^16^ and therefore very close to the range of test–retest variability. Indeed, the spread of residuals of the predictions in Figure 4, at least for the mesopic test, was compatible with the 95% test–retest variability range reported by Welker et al.^15^ and Pfau et al.^10^ Hence, although more complex modeling, such as with machine learning,^36^ might increase the accuracy of the prediction of functional loss, a large improvement is unlikely. Previous work reported better structure–function relationship in patients with AMD, but the results are not always directly comparable with ours and focused on mesopic tests. Querques et al.,^37^ for example, used a clinical categorical classification of autofluorescence patterns and included eyes with geographic atrophy. Acton et al.^38^ classified the tested locations based on the presence or absence of microperimetric defects and studied the structural differences between these two classes. Hence, in contrast to our work, the structural metric was used as the independent variable in their analysis. Work by Wu et al.^16^ was more similar to ours and also reported a better predictive power of their structure–function model for mesopic tests. However, their model explicitly included age and eccentricity and predicted the raw sensitivity. Given the very small sensitivity loss in these patients, the parameters of age and eccentricity are likely to be stronger predictors than structural features. Moreover, the effect of eccentricity was modeled with separate regression equations, one for each location, on a limited number (five) of retinal positions from the fovea. Such modeling relied on the assumption that the same location in the perimetric grid was at the same eccentricity from the fovea across different tests. Such an assumption was not possible in our case, since we positioned the testing grid on the structural maps by matching the fundus pictures from the MAIA and the Spectralis acquisitions. Therefore, the position of the grid could vary from test to test in relation to the anatomic fovea. Finally, if eccentricity was instead included as a continuous variable, such an approach would have required very different structure–function models for each microperimetric test, because of the large differences in the effect of eccentricity (Fig. 3). Instead, in our analysis, age and eccentricity were part of the normative models used as a reference to calculate the sensitivity loss in patients with drusen, so that the contribution of the structural features could be isolated. Therefore, we feel that our methodology yields results that are more reflective of the actual effect of structural changes in patients with drusen. Nevertheless, the modeling proposed by Wu et al.^16^ would be extremely valuable if such limitations could be overcome by controlling the position of the perimetric grid. For example, OCT maps could be used to center the perimetric grid on the anatomic fovea across different tests and standardize its position on the retina. This could be the objective of future work.

In isolation, PRR was the best-performing structural predictor of sensitivity loss for all tests (Table 2), but predictive power was low. Reflectivity of the outer layers was also an important parameter in previous structure–function analyses in patients with drusen.^16^^,^^38^^,^^39^ Compared to those reports, which used a simple qualitative score to classify the integrity of the outer layers, we developed a novel method that allows for an objective and quantitative mapping of this feature. We also performed our calculations on the raw OCT signal rather than the transformed images, which allowed for easy linear computations that more precisely reflect the optical properties of the retinal layers.^40^^,^^41^ One limitation of this method is that it does not account for hyperreflective foci, which can be indicative of more advanced damage to the outer retina.^42^ The explanations for the statistically significant effect of PRR can be multiple. Altered signal from the outer segments can be an indicator of photoreceptor atrophy^43^^,^^44^ and therefore justify our findings. However, optical effects might also play a role. When the RPE is distorted by the presence of drusen, the photoreceptors change their alignment with respect to the optical axis of the eye. Photoreceptors are known to interact directionally with light. This directional preference, often referred to as the Stiles-Crawford effect, has been shown to affect both reflectivity in OCT imaging^45^^,^^46^ and retinal sensitivity.^45^ Therefore, it could be a reason for the weakly concordant structural and functional changes in patients with drusen. If this was the only explanation, however, all the relevant information should be captured by the RPE-E (i.e., the changes in the geometry of the photoreceptor layer). Instead, this parameter was a significant predictor in both the mesopic and the scotopic cyan test even in the multivariable analysis. Moreover, the correlation between RPE-E and PRR, while significant, was weak. The strongest (negative) correlation between structural parameters was instead found for RPE-E and GAF. The appearance of autofluorescence in drusen can be varied and depends on the composition of the subretinal material and the status of the overlying retina.^43^^,^^47^ In general, a reduction in FAF is thought to reflect a poorer health of the overlying photoreceptors and RPE cells.^43^^,^^47^^,^^48^ This hypothesis agrees with our results since GAF was a significant predictor also in the multivariate analysis and the only significant predictor for the scotopic red test, with lower GAF signal corresponding to lower sensitivity.

**Table 2.** *R* ^2^ and *P* Values for the Univariable Structure–Function Correlations

	*R* ^2^ (*P* Value)		
Characteristic	Mesopic	Scotopic, Red	Scotopic, Cyan
RPE-E	0.018 (*P* < 0.001)	0.001 (*P* = 0.005)	0.013 (*P* < 0.001)
PRR	0.058 (*P* < 0.001)	0.024 (*P* = 0.006)	0.069 (*P* < 0.001)
GAF	0.022 (*P* < 0.001)	0.005 (*P* < 0.001)	2 × 10^−4^ (*P* < 0.001)

One important limitation to the application of the proposed analysis is the time-consuming process to segment the retinal images, especially in the presence of drusen. However, advances in segmentation techniques^49^^,^^50^ might significantly contribute to a more precise and quick quantification of relevant features in OCT images. Moreover, the choice of the OCT image section to measure the PRR was empirically based on preliminary calculations. A more refined definition of its boundaries might make the calculations more robust. Other limitations were the small sample size for the group of interest (eyes with drusen) and the use of only cross-sectional data. Eyes in the data set could also contain a mixture of Subretinal Drusenoid Deposits (SDDs) and drusen. In fact, eyes with pseudodrusen were identified only using fundus images, regardless of their appearance on the OCT. This was mainly due to the difficulty of reliably distinguishing SDDs from drusen on the raster scans. Finally, our definition of healthy eyes was mainly based on the presence of structural alterations on the OCT scans. Other data, such as intraocular pressure or traditional perimetry, were not available. However, no direct statistical comparison was performed between the healthy cohort and eyes with drusen. Any imprecision in the definition of the healthy cohort has therefore little bearing on our results. Lens opacity could also affect FAF imaging and microperimetric sensitivity. However, all eyes except for one with drusen had no or very mild lens opacity as quantified by the PNS (grades 0–1; see sample description). The only eye with a PNS = 2 did not show a reduced sensitivity compared to the rest of eyes with drusen. The use of systemic drugs was also recorded, although not in detail. The only class of drugs that could have interfered with the perimetric test were the antidepressants, used by only five healthy individuals and three patients with drusen.

Future work will focus on using this structure–function framework to improve the detection of functional progression with microperimetric tests in a longitudinal data set. The model could be employed as a scaling method to convert the structural measurements into its functional equivalent. The progression rate calculated on the converted structural measurements could then be used as prior knowledge in a Bayesian framework to calculate the progression rate of the actual functional measurements. A similar approach has proven effective in other contexts,^51^ making the fitting results more robust to the intrinsic variability within the perimetric test. Moreover, customized microperimetric grids could be used to improve the detection of damage by directly sampling over locations affected by drusen. Finally, the same methodology could be applied to a data set enriched with eyes with pseudodrusen and SDDs to accurately investigate the structure–function features of this subgroup.

## Figures and Tables

**Figure A1.**
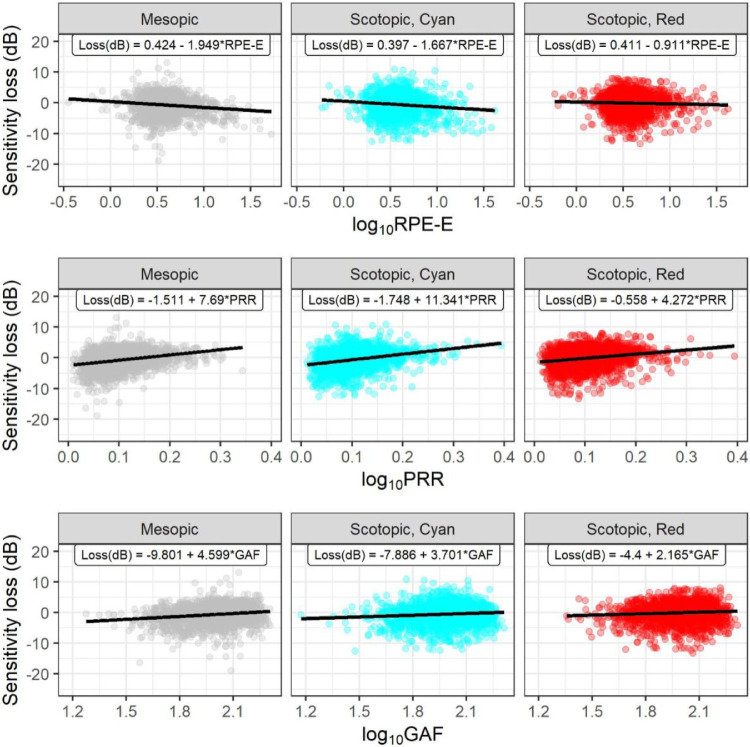
Univariable structure–function models. The equations for the relationship are reported inside the graphs. In the equations, all structural predictors are assumed log_10_ transformed. The asterisk represents the product sign.

## Appendix

Figure A1 shows the univariable structure–function relationship for the different structural parameters.

Table A1 reports both the full-interaction and additive-only versions of the multivariable models. For fairness of comparison, the full-interaction models were used for all calculations, since the interactions significantly improve the fit for the scotopic tests. However, for the mesopic test, most of the interactions were not significant. A simpler model could be achieved by stepwise selection using the Akaike information criterion.^34^ The optimal model for the mesopic test is reported in Table A2^5^. Additive-only models are reported for ease of interpretation of the linear effect of each predictor on sensitivity loss. All predictors were transformed in log_10_ scale. Table A3 reports the Beckman classification used in this study.

**Table A1.** Coefficients, Reported as Estimate (Standard Error), for the Predictors in the Multivariable Models

	Test Estimate (Standard Error)					
Variable	Mesopic	*P* Value	Scotopic, Red	*P* Value	Scotopic, Cyan	*P* Value
Full-interaction model						
Intercept	–3.047 (4.208)	0.469	–13.373 (5.36)	0.013	–17.504 (4.968)	<0.001
PRR	–49.741 (41.579)	0.232	98.794 (52.526)	0.060	158.915 (48.742)	0.001
RPE-E	–13.659 (7.261)	0.06	19.47 (8.704)	0.025	13.229 (8.055)	0.101
GAF	1.657 (2.126)	0.436	6.84 (2.687)	0.011	8.629 (2.489)	0.001
PRR × RPE-E	131.792 (71.839)	0.067	–202.157 (88.295)	0.022	–221.883 (81.945)	0.007
PRR × GAF	23.551 (20.892)	0.260	–49.594 (26.274)	0.059	–78.312 (24.372)	0.001
RPE-E × GAF	5.511 (3.706)	0.137	–10.458 (4.396)	0.017	–7.766 (4.069)	0.056
PRR × RPE-E × GAF	–58.159 (36.268)	0.109	105.098 (44.242)	0.018	118.029 (41.077)	0.004
Additive-only model						
Intercept	–8.268 (0.996)	<0.001	–3.84 (1.142)	0.001	–6.976 (1.107)	<0.001
PRR	5.985 (1.362)	<0.001	3.585 (1.559)	0.022	9.994 (1.529)	<0.001
RPE-E	–1.41 (0.277)	<0.001	–0.618 (0.333)	0.063	–1.107 (0.329)	0.001
GAF	3.916 (0.462)	<0.001	1.87 (0.529)	<0.001	3.03 (0.512)	<0.001

**Table A2.** Coefficients, Reported as Estimate (Standard Error), for the Predictors in the Multivariable Optimal Model for the Mesopic Test, Selected through the Akaike Information Criterion

	Test-Estimate (Standard Error)	
Optimal Model	Mesopic	*P* Value
Intercept	–7.317 (1.02)	<0.001
PRR	–3.196 (2.67)	0.231
RPE-E	–2.885 (0.461)	<0.001
GAF	3.835 (0.461)	<0.001
PRR × RPE-E	17.304 (4.335)	<0.001

**Table A3.** Beckman Classification Used to Grade the Color Fundus Pictures of the Participants

Numerical Code	AMD Classification	Stage Definition (Areas of Lesions within 2 Disc Diameters from the Foveal Center)
Beckman group 0	No obvious aging changes	No drusen—no pigmentary changes*
Beckman group 1	Normal aging changes	Only drusen ≤63 µm—no AMD pigmentary abnormalities*
Beckman group 2	Early AMD	Medium drusen >63 µm and ≤125 µm—no AMD pigmentary abnormalities*
Beckman group 3	Intermediate AMD	Large drusen >125 µm—any other AMD pigmentary changes*
Beckman group 4	Late AMD	Neovascular AMD and/or any geographic atrophy
